# Exploiting the Close-to-Dirac Point Shift of the Fermi Level in the Sb_2_Te_3_/Bi_2_Te_3_ Topological Insulator Heterostructure for Spin-Charge Conversion

**DOI:** 10.1021/acsami.3c08830

**Published:** 2023-10-20

**Authors:** Emanuele Longo, Lorenzo Locatelli, Polychronis Tsipas, Akylas Lintzeris, Athanasios Dimoulas, Marco Fanciulli, Massimo Longo, Roberto Mantovan

**Affiliations:** † CNR-IMM, Unit of Agrate Brianza, Via C. Olivetti 2, Agrate Brianza 20864, Italy; ‡ National Centre for Scientific Research “Demokritos”, Institute of Nanoscience and Nanotechnology, Agia Paraskevi 15341, Athens, Greece; § Department of Material Science, University of Milano Bicocca, Via R. Cozzi 55, Milan 20125, Italy; ∥ Department of Chemical Science and Technologies, University of Rome Tor Vergata, Via della Ricerca Scientifica, Rome 100133, Italy; # Department of Physics, School of Applied Mathematical and Physical Sciences, National Technical University of Athens, Athens 10682, Greece

**Keywords:** spintronics, topological insulators, Fermi level engineering, MOCVD, spin pumping, spin-charge conversion

## Abstract

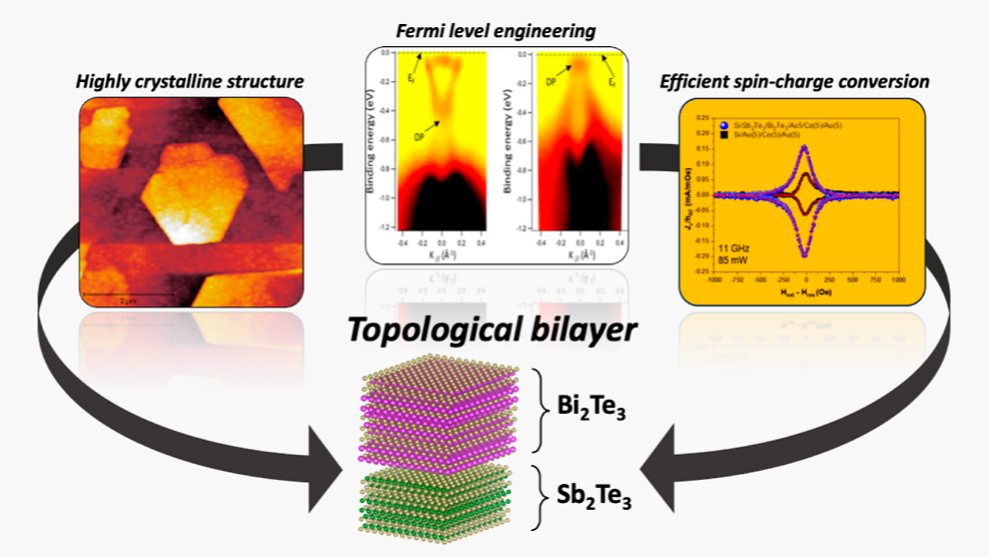

Properly tuning the Fermi level position in topological insulators is of vital importance to tailor their spin-polarized electronic transport and to improve the efficiency of any functional device based on them. Here, we report the full in situ metal organic chemical vapor deposition (MOCVD) and study of a highly crystalline Bi_2_Te_3_/Sb_2_Te_3_ topological insulator heterostructure on top of large area (4″) Si(111) substrates. The bottom Sb_2_Te_3_ layer serves as an ideal seed layer for the growth of highly crystalline Bi_2_Te_3_ on top, also inducing a remarkable shift of the Fermi level to place it very close to the Dirac point, as visualized by angle-resolved photoemission spectroscopy. To exploit such ideal topologically protected surface states, we fabricate the simple spin-charge converter Si(111)/Sb_2_Te_3_/Bi_2_Te_3_/Au/Co/Au and probe the spin-charge conversion (SCC) by spin pumping ferromagnetic resonance. A large SCC is measured at room temperature and is interpreted within the inverse Edelstein effect, thus resulting in a conversion efficiency of λ_IEEE_ ∼ 0.44 nm. Our results demonstrate the successful tuning of the surface Fermi level of Bi_2_Te_3_ when grown on top of Sb_2_Te_3_ with a full in situ MOCVD process, which is highly interesting in view of its future technology transfer.

## Introduction

The use of topological matter for spintronics has been attracting huge interest since it paves the way toward highly energy-efficient and ultrafast devices.^1−3^ In particular, topological insulators (TIs) represent a very interesting case due to their unconventional electronic band structure.^4,5^ In principle, TIs are characterized by insulating bulk states (BS) and by linearly dispersed (i.e., Dirac-like) highly conductive topological surface states (TSS). A useful aspect of the TSS is their helical spin polarization arising from the so-called spin-momentum locking, where the spin of the electrons moving along a specific direction is locked orthogonally to their motion.^4^ Recently, TIs have been proposed as promising candidates to achieve efficient data read-out in the MESO devices proposed by Intel for future processing-in-memory architectures.^1,6^ TIs are of particular interest in implementing spintronic devices that are based on spin-charge conversion (SCC) mechanisms.^7,8^ The reciprocal effect, i.e., charge-spin conversion (CSC), has also been demonstrated at the interface between TIs and ferromagnetic (FM) layers, showing the great potentiality of TIs when compared to traditional heavy elements for spin–orbit torque-based devices.^9,10^

However, most of the 3D-TIs, such as Bi_2_Se_3_, Bi_2_Te_3_, and Sb_2_Te_3_, have their Fermi level (*E*_F_) cutting the conduction or valence band. Therefore, the electrical conduction in TIs is often characterized by the superposition of conduction mechanisms originating from the TSS and the BS, the latter being detrimental to the optimization of both SCC and CSC in TIs-based devices.^11^ In the specific case of Bi_2_Te_3_, few reports exist discussing its use for SCC applications, mostly conducted at a low temperature.^12^

Several strategies have been proposed to engineer the position of the *E*_F_ so as to adjust it ideally at the crossing of the linearly dispersed bands of the TSS, i.e., close to the Dirac point (DP). One of the most widely attempted strategies is to control the TIs’ stoichiometry by varying the molar fraction of the constituting elements. In the case of chalcogenide-based 3D-TIs, a notable example is given by the (Bi_1–*x*_Sb_*x*_)_2_Te_3_ family, for which Su et al.,^13^ Okada et al.,^14^ and Kondou et al.^15^ have demonstrated how fine control of the *E*_F_ position can be achieved by gradually changing the Sb content. In these works, the authors showed an enhanced SCC efficiency for a specific Sb concentration, where the Fermi level is rigidly shifted in the proximity of the DP. An alternative strategy consists of controlling the chemical doping of the TIs’ surfaces. In fact, Hsieh et al.^16^ demonstrated that the modification of the Ca content in Bi_1–*x*_Ca_*x*_Te_3_ compounds can increase the surface hole-donor concentration, which progressively pushes the *E*_F_ position toward the DP. Similarly, the use of interlayers between TIs and neighboring FM layers can also be used to intentionally engineer the band structure of a TI, finally enhancing the SCC efficiency in TI/FM heterostructures. For instance, Sun et al.^17^ reported in 2019 a study on the SCC mechanisms in Bi_2_Se_3_/Bi/FM systems as a function of the Bi thickness. As a result, they have demonstrated the possibility to deeply modify the system, generating a Rashba-like 2D electronic gas band structure superimposed on the TSS of a Bi_2_Se_3_ layer, hence achieving full control of the SCC efficiency of the proposed heterostructure. The direct modification of the chemistry of a material is not the only way to engineer the Fermi level position. Interestingly, Du et al.^18^ have predicted that the internal strain present in a topological material (i.e., antiferromagnetic TI) can modify the position of the Fermi level due to the presence of defects and substitutional dopants in the structure.

An alternative strategy to move *E*_F_ toward the DP without chemically changing the nature of the TIs and without the use of voltage gating is to combine p and n-type materials. Eschbach et al. have presented the first effort of making a Bi_2_Te_3_/Sb_2_Te_3_ “p–n junction” by MBE, in which the *E*_F_ position of the top Sb_2_Te_3_ TI has been observed to change, depending on its thickness, with the *E*_F_ cutting the DP for 15 QL of Sb_2_Te_3_.^19^ In line with this first experiment, others have followed, in which MBE has been employed to fabricate Bi_2_Te_3_/Sb_2_Te_3_ p–n junctions, where Sb_2_Te_3_ has always been chosen as the top TI.^20,21^

In our previous work,^11^ we investigated the topological properties of Bi_2_Te_3_ thin films grown by metal organic chemical vapor deposition (MOCVD) on 4″ Si(111) substrates, demonstrating the presence of TSS lying in the bulk insulating gap of the material. Through angle-resolved photoemission spectroscopy (ARPES) measurements, the Dirac-like dispersion of such surface states has been established, despite the position of the Fermi level being found to be within the conduction band of the material, as typically observed in Bi_2_Te_3_. By performing magnetotransport experiments, a clear, weak antilocalization effect has been observed in Bi_2_Te_3_ and attributed to a 2D-type of conduction.^11^ However, a remarkable contribution from the BS to the Bi_2_Te_3_ transport has still been evidenced, thus hindering the ideal contribution from the TSS. Indeed, it has already been shown by Wu et al.^22^ that the presence of BS at the Fermi level is a competitive transport mechanism which reduces the SCC performance of a TI, inhibiting pure quantum topological transport through the TSS.

In the wake of these considerations and inspired by previous attempts in making Bi_2_Te_3_/Sb_2_Te_3_ p–n junctions,^19−21^ in the present manuscript, we showcase the possibility of engineering the *E*_F_ position in MOCVD-produced Bi_2_Te_3_ thin films when grown on top of Sb_2_Te_3_. To our knowledge, this is the first attempt at making a “reversed” Sb_2_Te_3_/Bi_2_Te_3_ p–n junction in which Bi_2_Te_3_ is on top. Moreover, the use of MOCVD could have advantages when compared to MBE in view of future technology transfer. In particular, the commensurate growth of a 90 nm thick Bi_2_Te_3_ layer on top of a nearly epitaxial Si(111)/Sb_2_Te_3_ seed substrate is conducted by optimizing a full in situ process, where the whole heterostructure is grown by MOCVD on Si(111) over an area up to 4″. The structural properties of the obtained samples are studied by X-ray diffraction (XRD) performed in Bragg–Brentano geometry to assess their crystalline quality. Atomic force microscopy (AFM) is employed for direct visualization of the surface morphology and the roughness estimation of the Bi_2_Te_3_ layer. ARPES measurements on the Sb_2_Te_3_/Bi_2_Te_3_ heterostructure are performed to probe the shifting of *E*_F_ in the Bi_2_Te_3_ top layer, and the obtained results are compared with those previously reported on the single Bi_2_Te_3_ layer directly deposited on Si(111).^11^ In order to correlate the band engineering with the functionality of the proposed heterostructure, the deposited topological bilayer is coupled with a Au(5 nm)/Co(5 nm)/Au(5 nm) FM trilayer grown by evaporation, and spin pumping FMR resonance (SP-FMR) measurements are performed to extract the SCC efficiency of the system. The measured SP-FMR signals are interpreted by adopting the inverse Edelstein effect (IEE) model, from which a remarkable IEE length λ_IEE_ ∼ 0.44 nm is extracted at room temperature for the Sb_2_Te_3_/Bi_2_Te_3_ heterostructure, this being a considerably high value within the class of chalcogenide-based 3D-TIs systems (see Table 1 of ref (23)). Here, the adoption of the Au interlayer turns out to be beneficial to protect the Bi_2_Te_3_ TSS from degradation, avoiding uncontrolled interfacial intermixing, as has also been proven for similar systems.^23−25^

## Results and Discussion

**Figure 1:**
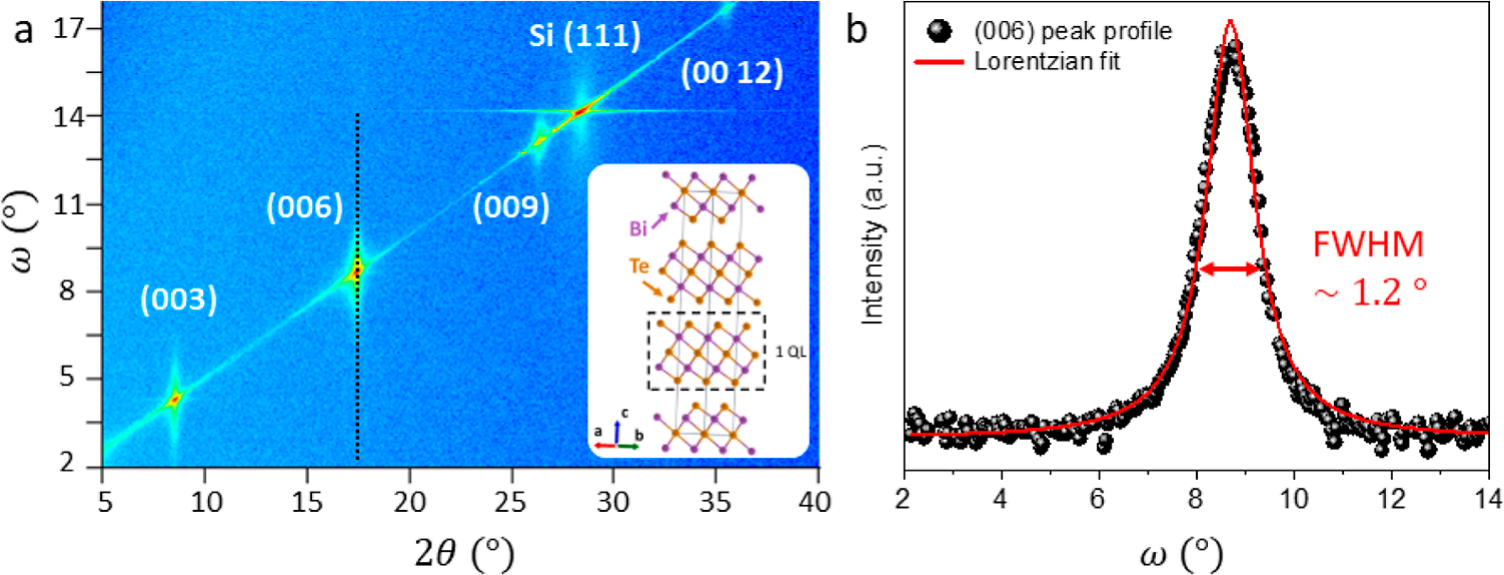
(a) XRD pattern acquired in the Bragg–Brentano geometry. The more intense signals (red elongated dots) indicate the OOP orientation of the Bi_2_Te_3_ crystalline planes [inset: Bi_2_Te_3_ crystalline structure drawn with the VESTA software,^26^ according to the ICSD code 74348 and the definition of QL]. (b) Rocking angle profile of the (006) reflection [dotted line in panel (a)]. The acquired data are fitted with a Lorentzian curve to extract the value of the mosaicity for the OOP-oriented crystals, which corresponds to the FWHM of the peak.

In Figure 1a the XRD pattern of the Si(111)/Sb_2_Te_3_/Bi_2_Te_3_ heterostructure acquired in the Bragg–Brentano geometry is depicted, and the inset shows the Bi_2_Te_3_ crystalline structure drawn with the VESTA software.^26^ Through this measurement, the out-of-plane (OOP) orientation of the crystalline grains of the rhombohedral Bi_2_Te_3_ layer belonging to the *R*3̅*m* space group is extracted.^27^ The only signals emerging from the contour plot shown in Figure 1a (red spots) correspond to the Bi_2_Te_3_ crystalline planes oriented along the [00*l*] direction, as expected for highly ordered crystalline structures. To directly quantify the deviation from the full OOP orientation of the (00*l*) planes with respect to the Si(111) surface, the mosaicity of the Bi_2_Te_3_ layer is calculated by extracting the rocking curve around the (006) reflection (black dotted line in Figure 1a). In Figure 1b the variation of the signal intensity as a function of the rocking angle ω is reported and fitted with a Lorentzian curve (red solid line), where the full width at half-maximum (FWHM) represents the mosaicity of the film, corresponding to (1.20 ± 0.02)°. Remarkably, such a low value is comparable with that of chalcogenide thin films deposited by physical methods (i.e., molecular beam epitaxy).^28^ The analysis of the Bragg–Brentano diffraction pattern and the quantification of the mosaicity suggest a nearly single-crystal fashion of the MOCVD-grown Bi_2_Te_3_ film.

**Figure 2:**
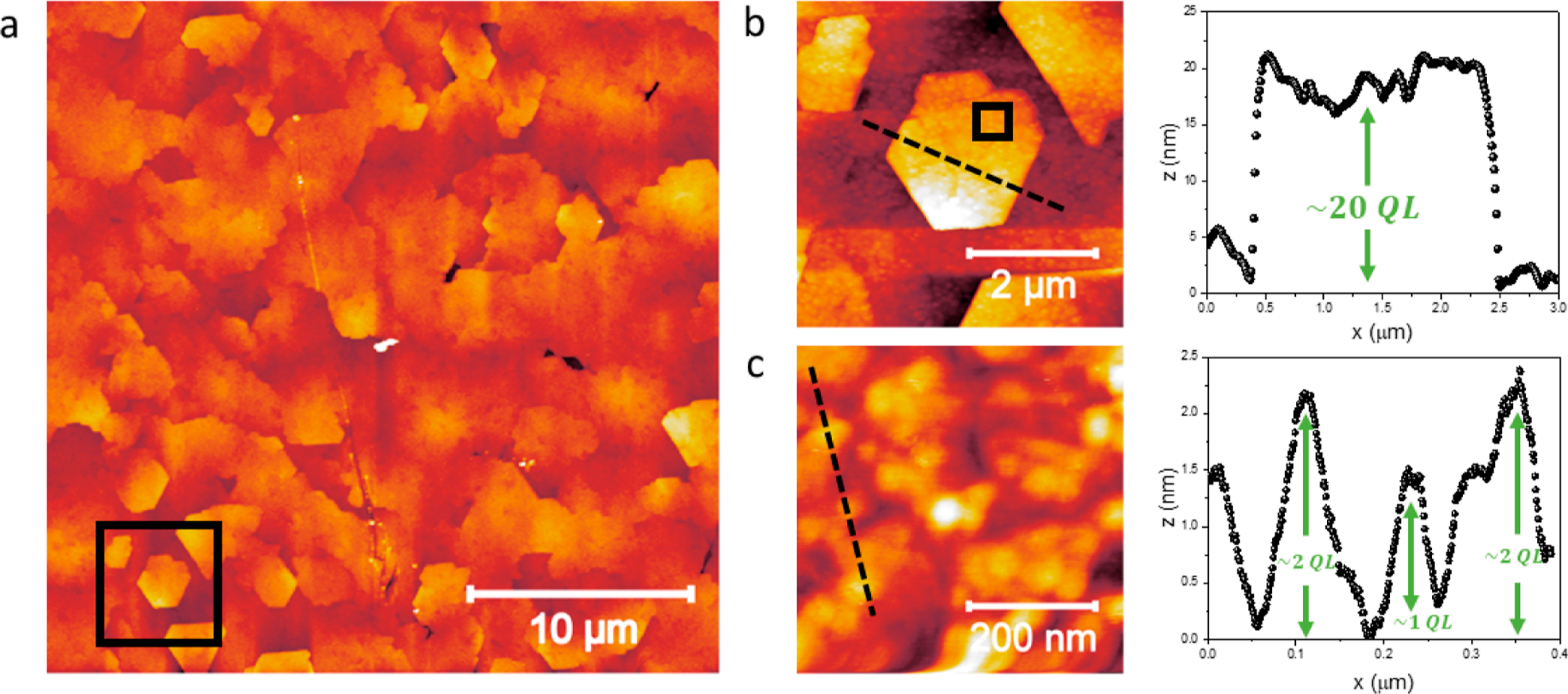
AFM measurement of the Bi_2_Te_3_ uncapped surface. (a) Scan area of 30 × 30 μm^2^, where the presence of several hexagon-like flakes is clearly visible. In the inset, a 5 × 5 μm^2^ scan of the area highlighted by the dashed black square in (a) is reported. The height profile of the Bi_2_Te_3_ flake is reported in panel (b). (c) Scan limited to a 0.5 × 0.5 μm^2^ area is shown. From panel (c), we measure a morphological *R*_RMS_ of 0.69 nm.

To provide further insights into the surface morphology of the Bi_2_Te_3_ layer, AFM measurements are conducted on different areas of the sample. In Figure 2a, an AFM acquisition on a 30 × 30 μm^2^ area of the Bi_2_Te_3_ bare surface is reported. Here, several in-plane-oriented Bi_2_Te_3_ hexagonal-like flakes are clearly visible, confirming the high crystalline nature of the deposited film, its crystalline symmetry, and a remarkable in-plane order.^27^

From the same picture, it turns out that some grains are merged in a more disordered fashion, a fact that does not always allow us to distinguish the shape of a single flake. This evidence is fully in accordance with the FWHM value extracted from the XRD analysis in Figure 1b, confirming the presence of Bi_2_Te_3_ regions with slightly disordered polycrystalline portions.

In Figure 2b, an AFM scan on a 5 × 5 μm^2^ smaller area is performed to highlight the flake geometry and to precisely extract its height profile, as shown by the black solid square in panel (a). The right side of Figure 2b shows the linear profile corresponding to the black dashed line in the left picture of panel (b), from which it turns out that the height of the selected flake is approximately 20 nm, thus being composed of about 20 Bi_2_Te_3_ quintuple layers (QLs).

To have an estimation of the surface roughness within a single Bi_2_Te_3_ flake, an additional scan is performed on a 0.5 × 0.5 μm^2^ area, as reported in Figure 2c. Here, a root main square roughness (*R*_RMS_) of 0.69 nm is extracted, a value which is sufficiently low to allow the possible integration of the MOCVD-grown Bi_2_Te_3_ with other materials, evidencing the very smooth morphology of the MOCVD-grown Bi_2_Te_3_ grains. However, the latter value is slightly higher than the one calculated for the bare Si(111)/Bi_2_Te_3_ reported in ref (27) for similar films, namely 0.5 nm.

The linear profiles acquired along the black dashed lines reported in Figure 2b,c show that the height of each step identifying a Bi_2_Te_3_ flake, or a smaller grain, is a multiple of a QL. Indeed, the Bi_2_Te_3_ structure is composed of QLs stuck together through van der Waals bonds (see inset of Figure 1a), demonstrating the very fine control of our MOCVD growth process.

**Figure 3:**
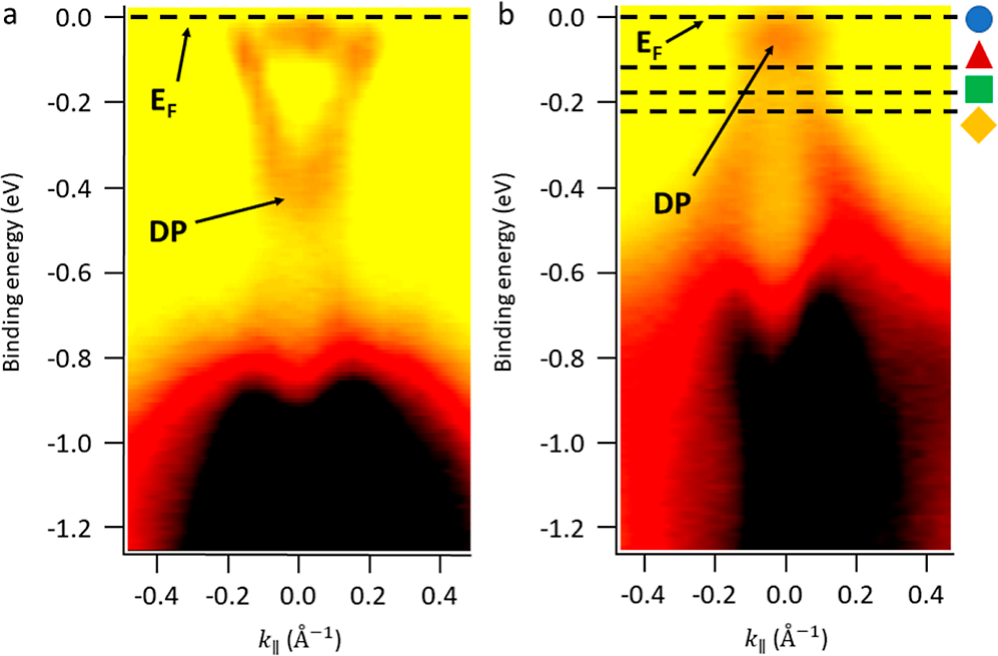
ARPES intensity map obtained for Si(111)/Bi_2_Te_3_, in panel (a), and Si(111)/Sb_2_Te_3_/Bi_2_Te_3_ heterostructures in panel (b). The black dashed lines in panel (b) indicate the different energies at which the contour plot is reported in Figure 4.

The ARPES technique is ideal for conducting direct visualization of the band structure of the Bi_2_Te_3_ layers closer to the film’s surface, Figure 3 shows a direct comparison between the band structure of Bi_2_Te_3_ when grown directly on Si(111) (Figure 3a),^11^ and on top of Sb_2_Te_3_ (Figure 3b). As ARPES is extremely sensitive to the first few surface layers, a cleaning procedure is conducted prior to measurements to remove the surface contaminants (see Methods and Supporting Information for details).

As it emerges from the ARPES image reported in Figure 3a, in the bare Si(111)/Bi_2_Te_3_ heterostructure, the Fermi level is clearly positioned across the conduction band of the material, influencing the overall electronic transport, as revealed by magnetotransport measurements reported in ref (11). On the other hand, by comparing this result with panel (b) of the same figure, the Fermi level is rigidly shifted within the bulk insulating gap when the Sb_2_Te_3_ seed layer is placed between the Si substrate and Bi_2_Te_3_, intercepting the TSS in the proximity of the DP.

By comparing the two panels of Figure 3, it can be noticed that the Bi_2_Te_3_ grown on top of the Sb_2_Te_3_ seed layer shows a rigid shift of the band structure with respect to the layer grown on top of Si(111). A similar observation has been reported by Pereira et al.^20^ for the reversed stack: Bi_2_Te_3_(bottom)/Sb_2_Te_3_(top), where the Sb_2_Te_3_ layer is subject to a modification of its band structure when just a few QLs are deposited, with a rigid shift of the chemical potential when a thicker layer is deposited. The latter condition is attributed to the different in-plane lattice constants of Sb_2_Te_3_ and Bi_2_Te_3_, being 0.42 and 0.44 nm, respectively. Such a 5% difference could induce an effective compressive (tensile) strain when Bi_2_Te_3_ is on top (bottom) to generate the observed chemical potential shift.

Even if we cannot exclude a slight change of the top Bi_2_Te_3_ thickness (within 10 nm in our estimation) when compared to the nominal 90 nm,^27,29^ this could hardly be the origin for the observed remarkable rigid shift of the *E*_F_ toward the DP that we observe (Figure 3), also according to recent literature reports.^30^

**Figure 4:**
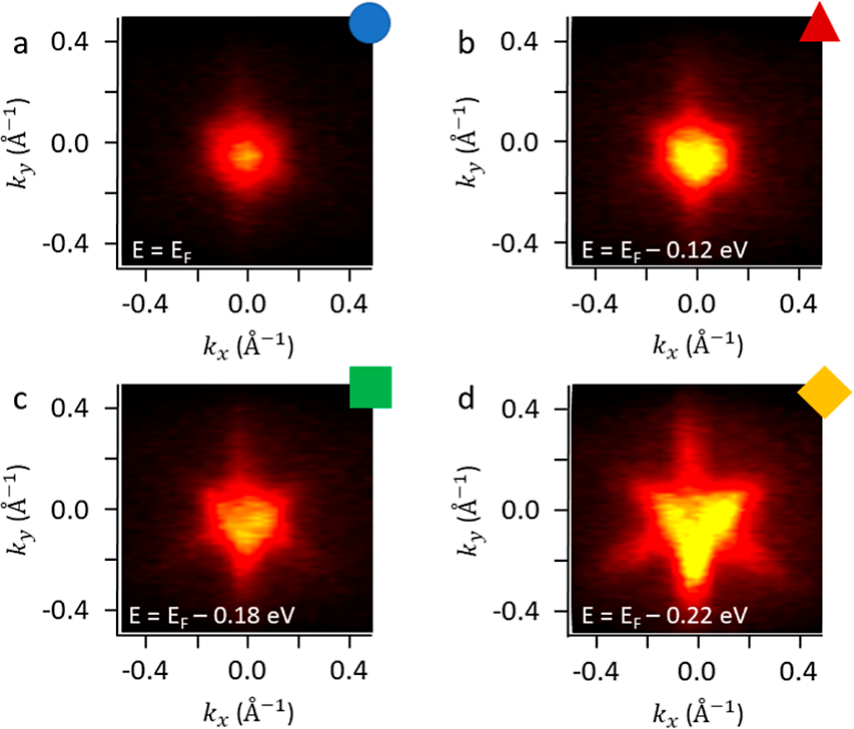
ARPES energy contour plots for the Si(111)/Sb_2_Te_3_/Bi_2_Te_3_ heterostructure. The K_*x*_ and K_*y*_ components are acquired along the K–G–K and M–G–M crystallographic directions in the *k*-space, respectively. The panels (a–d) marked with colored symbols indicate the various energy levels, as indicated in Figure 3b.

Different ARPES polar maps are acquired in this work at room temperature for the Si(111)/Sb_2_Te_3_/Bi_2_Te_3_ system at various binding energies, with the aim of following the evolution of the band structure from the surface to the BS of the Bi_2_Te_3_ top layer. In Figure 4, we report the ARPES contour plots for *E* = 0 (Fermi level), −0.12, −0.18, and −0.22 eV, as indicated by the colored symbols marked in panel (b) of Figure 3.

The pointwise symmetry of the contour plot in Figure 4a for *E* = *E*_F_ indicates that the system lies in the proximity of the DP, a condition which highlights the 2D quantum transport properties of the Si(111)/Sb_2_Te_3_/Bi_2_Te_3_ heterostructure. For energies lower than *E*_F_, the shapes of the spectra assume different geometries. At *E* = −0.12 eV, the hexagonal pattern appears, as expected for the symmetry rules of the TSS. Pushing down to *E* = −0.18 eV, a superposition between a hexagon and a triangular shape is observed, as a clear indication that the TSS and the BS are copresent at this energy. Finally, at *E* = −0.22 eV, the ARPES pattern is fully trigonal, therefore showing that the energy level is now solely crossing the Bi_2_Te_3_ BS.

The previous ARPES measurements represent a fundamental aspect of assessing whether the Si(111)/Sb_2_Te_3_/Bi_2_Te_3_ heterostructure possesses the prerequisites needed to exploit the topological transport in spintronic devices.

Having successfully moved the *E*_F_ close to the DP, it is now highly interesting to exploit the Bi_2_Te_3_ functionality for SCC by making use of SP-FMR. In SP-FMR, pure spin currents are generated in the FM layer and subsequently converted into charge currents flowing across the surface states of the TI.^23,31^ However, it is known that the direct contact between a FM and a TI can be detrimental for the TSS,^15,23,25^ so the adoption of appropriate nonmagnetic and low spin–orbit coupling interlayers is often beneficial to preserve the TSS and avoid chemical intermixing to successfully exploit SCC.^23−25^ We follow the same methodology as previously employed to probe SCC into Sb_2_Te_3_.^23,25^ In particular, we use Co as FM and Au as inter/capping-layers, with the following final stack structure (from the bottom): Si(111)/Sb_2_Te_3_/Bi_2_Te_3_/Au(5 nm)/Co(5 nm)/Au(5 nm). To properly estimate the SCC efficiency, the signals extracted by the functional heterostructure comprising the TI material are compared with a Si(111)/Au(5 nm)/Co(5 nm)/Au(5 nm) reference heterostructure prepared simultaneously during the same Au/Co/Au evaporation process. In the following, the Si(111)/Au/Co/Au and Si(111)/Sb_2_Te_3_/Bi_2_Te_3_/Au/Co/Au heterostructures are named S0 and S1, respectively.

**Figure 5:**
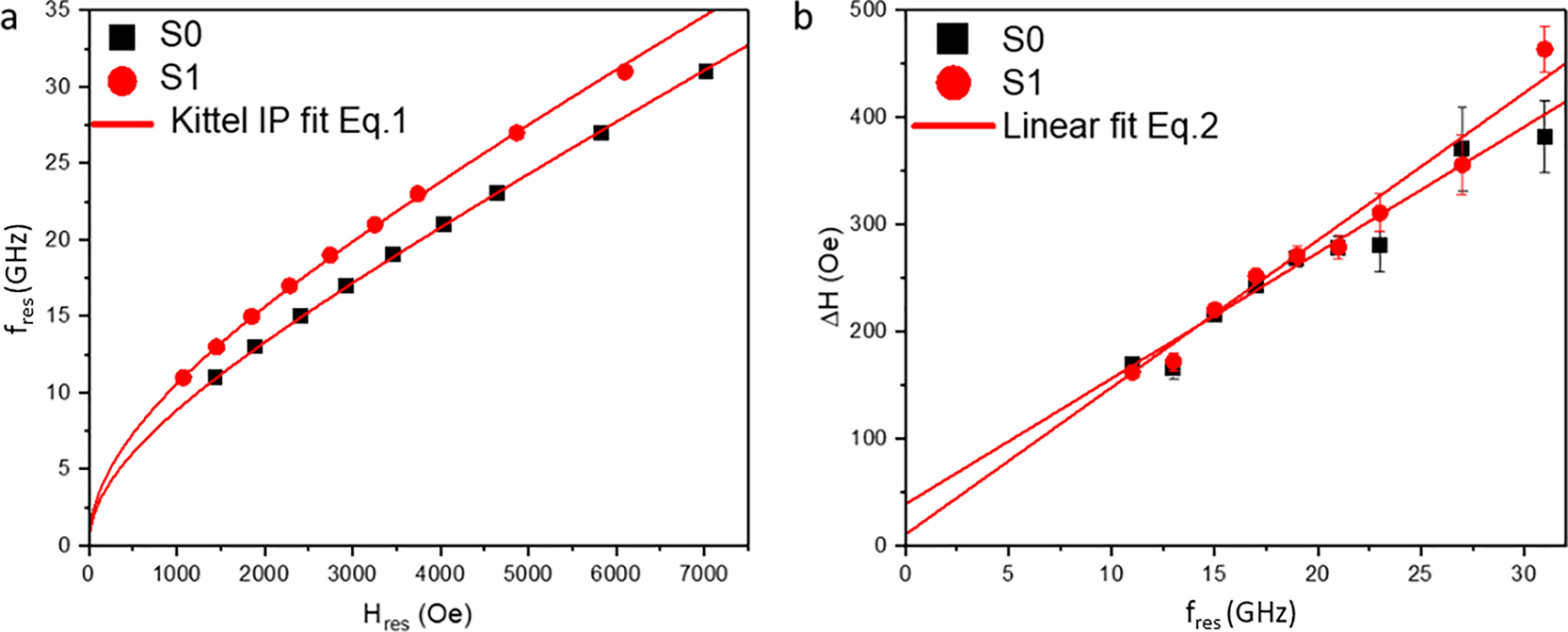
(a) Kittel dispersion for the IP configuration. The data set for the heterostructures S0 (black squares) and S1 (red circles) is reported and fitted with eq 1 (red solid line). (b) The signal line width as a function of the resonant frequency is reported for the same heterostructures of panel (a) and fitted with eq 2 (red solid line).

To extract the magnetization dynamics parameters necessary to quantify the SCC efficiency, broadband FM resonance (BFMR) measurements are performed.^23,32^ In Figure 5a, the Kittel curves acquired for heterostructures S0 and S1 are reported (black squares and red circles, respectively), where the resonant frequency (*f*_res_) is plotted as a function of the resonant magnetic field (*H*_res_) (see Methods). The evolution of the *f*_res_(*H*_res_) response is acquired over a large frequency range (11–30 GHz) to ensure reliable quantification of the parameters extracted from the fit with eq 1 (red solid line). For a polycrystalline FM thin film positioned in the in-plane (IP) configuration, the Kittel equation can be expressed as
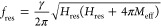
1where γ is the gyromagnetic ratio, *M*_eff_ is the effective magnetization, , where *e* and *m*_e_ are the charge and the mass of the electron, respectively, and *g* is the Landè *g*-factor, a quantity which links the electronic angular and spin momenta of a material.^33^

After extracting the *M*_eff_ value from eq 1, some information on the magnetic anisotropy can be achieved by writing the relation , where *M*_s_, *t*_Co_, *H*_k_, and *K*_s_ are the saturation magnetization, the thickness of the Co layer, the magnetic anisotropy field, and the surface magnetic anisotropy constant, respectively.

Adopting the same methodology as refs (23) and (25), from the fit of the Kittel dispersion reported in Figure 5a, we obtain  and . The extracted *g* values are very similar and perfectly in agreement with most of those found in the literature (*g* ∼ 2.25). The corresponding effective magnetizations are  and  for S0 and S1, respectively. These values are lower than the Co bulk value (),^34^ as typically observed in thin films, due to the possible presence of magnetic dead layers and/or magnetic shape anisotropy.^35^

In Figure 5b, the line width Δ*H* of the BFMR signal is plotted as a function of *f*_res_ and fitted according to the following eq 2.
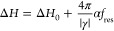
2where α represents the damping constant, and Δ*H*_0_ represents the inhomogeneous broadening. From the FMR theory,^33,36^ α accounts for how fast the magnetization vector *M* in a FM aligns along an applied external magnetic field at the resonance condition.^33^ According to the linear fit (red solid lines) of the data sets reported in Figure 5b, the values α^S0^ = (17.9 ± 1.3) × 10^–3^, Δ*H*_0_^S0^ = 38 ± 13 Oe, α^S1^ = (20.3 ± 1.2) × 10^–3^, and Δ*H*_0_^S1^ = 10 ± 12 Oe are extracted. Δ*H*_0_ provides information on the magneto-structural properties of the FM layer, accounting for sample imperfections or texturing. This value is acceptable and comparable for both samples, indicating a limited amount of magnetic disorder in the Co layer (i.e., magnetic dead layers, structural imperfections, etc.).

According to the spin pumping theory,^31,37^ the difference between the α values calculated for S0 and S1 is proportional to the real part of the spin mixing conductance Re(*g*_eff_^↑↓^) characterizing the interface between the FM and the adjacent spin-sink layer, in this case the Bi_2_Te_3_ layer. Re(*g*_eff_^↑↓^) is an intrinsic quantity of a system which accounts for the pure spin current flowing across the interface toward the Bi_2_Te_3_. In our case, by considering the measured Δα = (2.4 ± 2.5) × 10^–3^, we obtain Re(*g*_eff,Bi2Te3_^↑↓^) = 7.53 × 10^18^ m^–2^, a value perfectly in agreement with those extracted elsewhere for similar systems (see Table 1 ref (23)). For details about the procedure to extract the latter value, see the Supporting Information.

**Figure 6:**
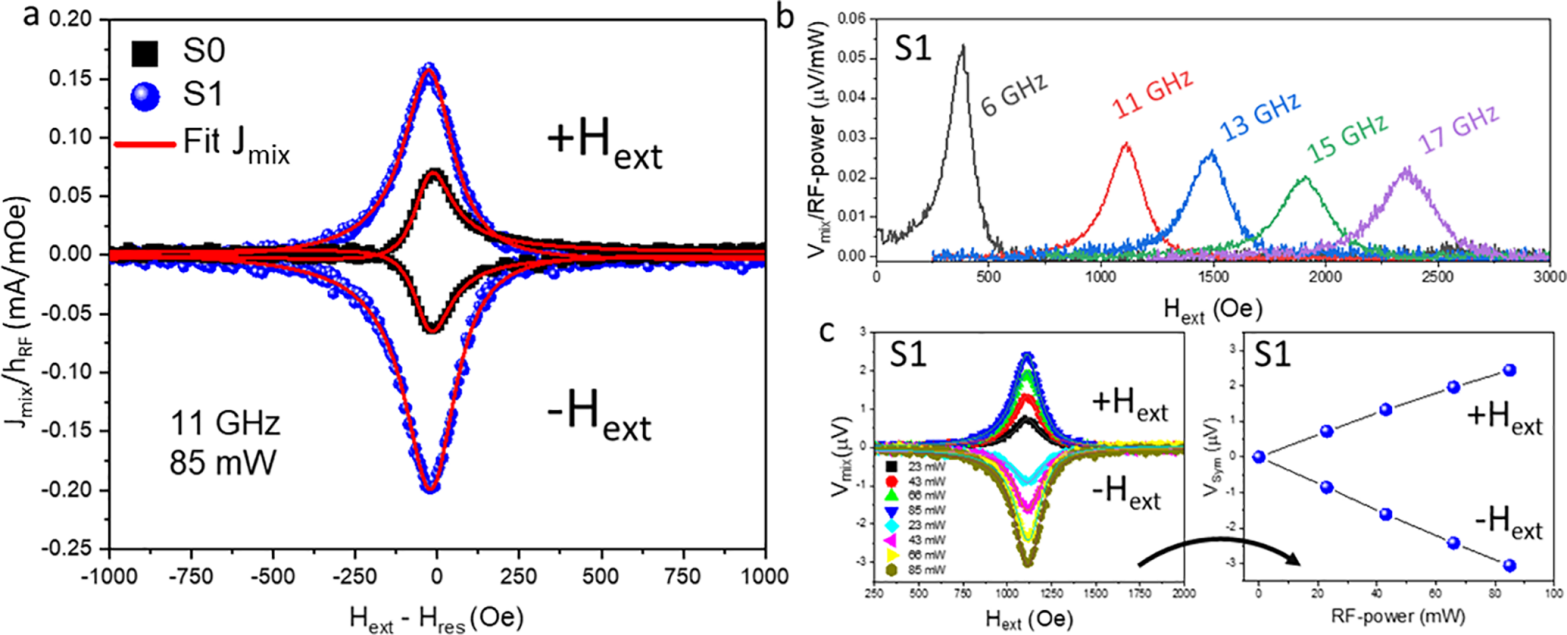
Electrically detected SP-FMR measurements. (a) 2D charge current generated in resonant condition and normalized to the strength of the oscillating magnetic field (*h*_RF_) for the heterostructures S0 (black squares) and S1 (blue circles) at a frequency of 11 GHz and 85 mW of RF power. The red solid line indicates a fit with eq 3. (b) Frequency-dependent response for the heterostructure S1. (c) (left) *V*_mix_ vs *H*_ext_ curves as a function of the RF power acquired at positive and negative applied external magnetic fields for heterostructure S1. (right) Dependence of the voltage symmetric component *V*_Sym_ as a function of the RF power for the curves reported in the left panel.

The enhancement of α alone is necessary but not sufficient evidence to conclude the existence of SCC, which can only be demonstrated by electrically detecting the spin pumping signal. In Figure 6, the electrically detected SP-FMR measurements carried out on the S0 and S1 heterostructures are shown. A detailed description of the experimental procedure and the theoretical background can be found both in ref (23) and partially in the Supporting Information.

In Figure 6a, the mixing charge current density *J*_mix_ is plotted for the heterostructures S0 (black squares) and S1 (blue circles), where the red solid lines indicate the fit of both curves, as obtained by the following Lorentzian function of eq 3.
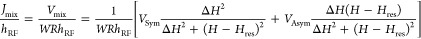
3where *h*_RF_, *W*, and *R* represent the transverse oscillating magnetic field, the width of the heterostructure, and its sheet resistance, respectively. In this case, the adopted values are *h*_RF_ = 0.73 Oe, *W*_S0_ = 2 mm, *W*_S1_ = 2 mm, *R*_S0_ = 16 Ω, and *R*_S1_ = 16 Ω (measured with a four-point probe). From such a fit, it is possible to isolate the symmetric component of the curve, which is proportional to the so-called symmetric voltage, *V*_Sym_, and directly connected to the spin pumping taking place at the TI/FM interface. Differently, the asymmetric part (∝*V*_Asym_) depends on spurious rectification effects (i.e., Oersted field, anisotropic magnetoresistance, etc.), thus being excluded from the estimation of the SCC mechanism.^38,39^ The curves in Figure 6a are highly symmetric, indicating that the rectification effects are not the main processes driving the electrical response in both heterostructures. Despite the similar shape of these curves, the intensity of the electrical signals generated from S1 is much higher than that in S0 at the same RF power, suggesting that the insertion of the Sb_2_Te_3_/Bi_2_Te_3_ bilayer is beneficial to generate a higher charge current density in S1 when compared to the reference S0. In order to remove a possible thermal component (i.e., Seebeck effect^40^) from the symmetric part of *J*_mix_, the actual signal arising from SP is obtained by mediating the symmetric part of the *J*_mix_ curves acquired for positive and negative magnetic fields, according to the equation . The extracted values are , , , and . For a quantitative estimate of the extra-current produced in S1 when compared to S0, we underline here the ratio , representing clear and direct evidence for the role played by the insertion of the Sb_2_Te_3_/Bi_2_Te_3_ heterostructure in generating a remarkable SCC. As a further confirmation that the SCC mechanism observed in S1 (Figure 6a) is consistent with the spin pumping effect, in Figure 6b the SP-FMR signals acquired for different RF frequencies are reported, demonstrating the relation between the measured voltage and the BFMR characteristic of the system. Moreover, the *V*_Sym_ component of the IEE signal should be linear when plotted as a function of the RF power, as shown in Figure 6c.^41^

In a spin pumping experiment, the SCC efficiency can be estimated mainly according to two models: the inverse spin-hall effect (ISHE) and the IEE. For the ISHE, the spin current generated into the FM layer is pumped and converted in the BS of the spin-sink layer (i.e., Bi_2_Te_3_), differently from the IEE, where solely the TSS are involved in the SCC. In our case, we performed several trials to measure the SCC in the interlayer-free Bi_2_Te_3_/Co heterostructure, but no significant SP was observed with respect to the reference heterostructure, as in the case of the Sb_2_Te_3_ TI previously investigated by some of us.^23,25^ Few works exist reporting the SCC effects in Bi_2_Te_3_ films directly in contact with the FM layer. For instance, in the very recent work of Liu et al.,^12^ the authors have observed an efficient spin current injection in the Bi_2_Te_3_/MnTe heterostructure only at *T* < 25 K, with a significant reduction around 100 K. Conversely, the introduction of interlayers at the Bi_2_Te_3_/FM interface is a reliable strategy to improve the SCC efficiency, as demonstrated by Li et al.^42^ adopting a BN/Al_2_O_3_ tunneling barrier.

The most plausible scenario in our case is that the Au interlayer is beneficial in protecting Bi_2_Te_3_’s TSS detected by ARPES (Figure 3), similarly to what has occurred in Sb_2_Te_3_.^23,25^ Therefore, the observed SCC can be represented in terms of the IEE, where the IEE diffusion length  is adopted as a figure of merit to determine the conversion efficiency, with *J*_s_^3D^ being the spin current density generated in the system (see Supporting Information). According to the parameters reported in Figure 6a and to the FMR response in Figure 5, we have *J*_s_^3D^ = 2.97 × 10^5^ A m^–2^, and therefore, . Despite our evidence being consistent with the IEE, the origin of the voltage signals in SP-FMR experiments is often highly debated (see Supporting Information).^43−45^ For the sake of clarity, a different fitting approach can be performed by fixing *g* ∼ 2.25 in eq 1 from the averaging of some values found in the literature for Co thin films. In this case, λ_IEE_ would be ∼0.40 nm, in strong accordance with the value reported above.

By comparing the λ_IEE_ extracted here with some of those found in the literature, we can infer that the 0.44 nm value calculated in our MOCVD-deposited heterostructure is a very large one (see Table 1 ref (23)). If we refer only to the SCC efficiency extracted from different chalcogenide-based topological compounds, it emerges that this value is among the highest reported so far at room temperature. Notably, TI-based systems to compare with are, for example, those reported by Mendes et al.^46^ with λ_IEE_ = 0.075 nm in (Bi_0.22_Sb_0.7_)_2_Te_3_, or λ_IEE_ = 0.28 nm in Bi_2_Se_3_ reported by Sun et al.,^17^ and recent works by some of us,^23,25^ showing that λ_IEE_ = 0.28 nm for a 30 nm thick Sb_2_Te_3_ epitaxial TI. Also, among the possible different interlayers, the choice of Au seems to be particularly favorable. As a comparison, in the work from He et al.,^47^ published in 2021, the authors exploited Ru and Ti interlayers in Bi_2_Te_3_/interlayer/CoFeB systems, extracting λ_IEE_ values ten times smaller than those we extract here.

## Conclusions

The need to properly tune the Fermi level position in TIs is of paramount importance to fully make use of their topologically protected spin-polarized electronic transport. In this work, we report a full in situ MOCVD process where highly crystalline 90 nm thick Bi_2_Te_3_ thin films are grown on top of epitaxial Sb_2_Te_3_ layers over 4″ Si(111) substrates. The use of Sb_2_Te_3_ as a seed layer turns out to be a valid strategy to shift the Bi_2_Te_3_’s Fermi level from the conduction band (as it occurs in single Bi_2_Te_3_ layers) to the insulating gap in very close proximity to the DP, as clearly visualized by room-temperature ARPES measurements. This generates ideally pure TSS, which we exploit in a simple spin-charge converter by conducting SP-FMR measurements. A remarkably high SCC efficiency is measured in the Si(111)/Sb_2_Te_3_/Bi_2_Te_3_/Au/Co/Au heterostructure, and by interpreting such a conversion within the IEE, we extract a high conversion efficiency of λ_IEE_ ∼ 0.44 nm at room temperature. Here, we demonstrate that the full in situ MOCVD growth of the Bi_2_Te_3_/Sb_2_Te_3_ heterostructure is successful in producing a TI characterized by ideal topologically protected surface states and that those states can directly be exploited for efficient SCC. This work paves the way for the future adoption of chemically deposited Bi_2_Te_3_ thin films on a large area Si wafer, thanks to the highly efficient SCC achieved via Fermi-level engineering.

## Materials and Methods

A nominally 90 nm thick Bi_2_Te_3_ layer^27,29^ is grown on a 4″ Si(111)/Sb_2_Te_3_(30 nm) wafer by means of MOCVD. Prior to the deposition of Sb_2_Te_3_, the Si(111) substrate is treated by means of HF acid to remove the native oxide, and an annealing process is performed to properly reconstruct the Si surface. Following the substrate conditioning, the growth of the seed layer of Sb_2_Te_3_ is performed at room temperature for 90 min and at a pressure of 15 mbar. The deposition is then followed by post-growth annealing, which is needed to promote further crystallization. Without removing the sample from the reactor, the chamber is brought to 350 °C, and the deposition of the layer of Bi_2_Te_3_ is performed at 75 mbar for 190 min. The growth parameters of the two TI layers are kept unchanged with respect to those employed to grow the Sb_2_Te_3_ and Bi_2_Te_3_ single layers on top of Si(111), as reported in refs (27) and (29).

The Si(111)/Sb_2_Te_3_/Bi_2_Te_3_ heterostructures dedicated to the SP-FMR studies are cut into smaller pieces and transferred into an Edwards Auto306 e-beam evaporation tool, together with the Si(111) reference substrates. A simultaneous deposition of the Au(5 nm)/Co(5 nm)/Au(5 nm) trilayer is conducted for all the heterostructures. The Si(111) substrates used as references are cleaned with isopropyl alcohol and treated with HF prior to the evaporation processes.

The Bragg–Brentano XRD pattern is acquired by an HRXRD IS2000 diffractometer equipped with a Cu K_α_ radiation source (λ = 1.5406 Å), a four-circle goniometer, and a curved 120° position-sensitive detector (Inel CPS-120). This configuration allows the detection of the asymmetric reflections produced by the crystalline planes not perfectly parallel to the sample surface, giving access to the value of the mosaicity of the crystalline grains composing the material.

AFM images are obtained using a Bruker dimension edge instrument operating in the tapping mode and using a sharp silicon AFM probe (TESPA, Bruker) with a typical radius of curvature in the 8–12 nm range. A polynomial background correction is applied to the raw data. To quantify the morphological surface, the root mean square roughness (RMS roughness, *R*_q_) value is adopted and expressed in nanometers. The different AFM images reported in the main text represent three independent measurements acquired on 30 × 30 μm^2^, 5 × 5 μm^2^, and 0.5 × 0.5 μm^2^ areas.

ARPES spectra are acquired at room temperature with a 100 mm hemispherical electron analyzer equipped with a 2D CCD detector (SPECS). The He I (21.22 eV) resonant line is employed to excite photoelectrons, yielding an energy resolution of 40 meV. The spot of the employed ARPES facility has an elliptical shape with an area of about 4 × 6 mm^2^. Thanks to this, the ARPES characterization conducted on our samples makes it possible to extract information about the dispersion of the material band structure in a relatively macroscopic area. ARPES is performed ex situ, so a two step surface cleaning procedure is needed to remove the oxidized species and contaminants, namely surface ion sputtering (Ar^+^ at 1.5 keV) and an annealing treatment at 270 °C. Subsequently, the film surface is probed with reflection high-energy electron diffraction and X-ray photoemission spectroscopy to verify the effectiveness of the treatments (see Supporting Information).

BFMR is performed using a broadband Anritsu-MG3694C power source (1–40 GHz), connected to a grounded coplanar waveguide, where the samples are mounted in a flip-chip configuration (the FM film is located close to the GCPW surface) with a 75 μm thick Kapton foil stacked in between to prevent the shortening of the conduction line. The sample-GCPW system is positioned between the polar extensions of a Bruker ER-200 electromagnet, maintaining its surface parallel to the external magnetic field *H*_ext_, in the so-called in-plane (IP) configuration. During the measurements, an RF current at a fixed frequency is carried toward the GCPW, and the transmitted signal is directed to a rectifying diode, converting the RF signal into a continuous DC current, subsequently detected by a lock-in amplifier downward the electronic line. The same instrumentation is adopted to conduct SP-FMR measurements. Here, the edges of the sample are in contact with Ag paint and connected to a nanovoltmeter. A DC voltage is detected under resonant conditions, fixing the RF frequency and power.

## Supporting Information Available

Grazing Incidence XRD on Si(111)/Sb_2_Te_3_/Bi_2_Te_3_; estimation of the spin mixing conductance and spin current density from FMR experiments; alternative calculation to estimate the SCC efficiency according to the Inverse Spin Hall Effect (ISHE); and XPS and RHEED measurements of the Si(111)/Sb_2_Te_3_/Bi_2_Te_3_ surface (PDF)
