# Supplementary material for: Exploiting the Close-to-Dirac Point Shift of the Fermi Level in the Sb2Te3/Bi2Te3 Topological Insulator Heterostructure for Spin-Charge Conversion
Source: ACS Appl Mater Interfaces. 2023 Oct 20;15(43):50237–45. doi: 10.1021/acsami.3c08830 (PMC10623560; doi:10.1021/acsami.3c08830)
Supplement: Supplementary file 1 — am3c08830_si_001.pdf [file am3c08830_si_001.pdf]

Supporting Information

Exploiting the close-to-Dirac point shift of Fermi level in Sb_2Te_3/Bi_2Te_3 topological insulator heterostructure for spin-charge conversion

Emanuele Longo[1,†] , Lorenzo Locatelli[1,†], Polychronis Tsipas[2], Akylas Lintzeris[2,3], Athanasios Dimoulas[2], Marco Fanciulli[4], Massimo Longo[1, 5], Roberto Mantovan[1,*]

[1]CNR-IMM, Unit of Agrate Brianza, Via C. Olivetti 2, 20864 Agrate Brianza, Italy

[2]National Centre for Scientific Research "Demokritos", Institute of Nanoscience and Nanotechnology, Agia Paraskevi, 15341, Athens, Greece

[3]Department of Physics, National Technical University of Athens, School of Applied Mathematical and Physical Sciences, Athens, Greece

[4]Department of Material Science, University of Milano Bicocca, Via R. Cozzi 55, Milan 20125, Italy

[5]Department of Chemical Science and Technologies, University of Rome Tor Vergata, Via della Ricerca Scientifica, 100133 Rome, Italy

[†]Equally contributed to the work. *roberto.mantovan@cnr.it

1. Grazing Incidence X-ray Diffraction on Si(111)/Sb_2Te_3/Bi_2Te_3

In Figure S1 the GIXRD pattern collected for the Si(111)/Sb_2Te_3/Bi_2Te_3 heterostructure is reported. The measurement is performed by fixing the rocking angle ω at 1°. The Miller indices for the Bi_2Te_3 crystalline structures are taken according to the file with the code 74348 of the ICSD database. The only visible peaks are the (003) and (006) reflections, which have a very low intensity. This condition indicates a residual

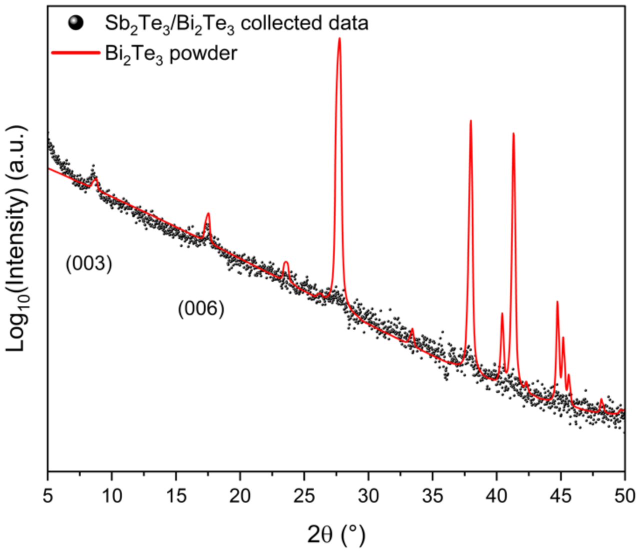

Figure S1. GXRD measurement on the Si(111)/Sb_2Te_3/Bi_2Te_3 heterostructure.

texturization of the film along the [00ℓ] direction, a typical condition for similar chalcogenide-based compounds grown on Si(111) substrates.[1–3] No further relevant peaks are present in the XRD pattern, a demonstration of the very high quality of the Si(111)/Sb_2Te_3/Bi_2Te_3 crystalline structure.

2. Estimation of the spin mixing conductance and spin current density from FMR experiments

From BFMR experiments it is possible to extract important information about the magnetization dynamic in a TI/FM system. A fundamental quantity defining the amount of spin current crossing the TI/FM interface is called spin mixing conductance ($g_{eff}^{\uparrow\downarrow}$). To calculate the value of $g_{eff}^{\uparrow\downarrow}$ for heterostructure S1, as reported in the main text, we use the following procedure.

By plotting the $\Delta H(f_{res})$ curve for the heterostructures S0 and S1, the α values for the two heterostructures are extracted by fitting the dataset with the equation:

$$\Delta H = \Delta H_0 + \frac{4\pi}{|\gamma|} \alpha\, f_{res} \qquad (1)$$

According to the SP theory,[4–6] the difference between the damping constant of heterostructures S1 and S0 is proportional to $g_{eff}^{\uparrow\downarrow}$, which can be calculated using the following relation:

$$\alpha_{FM/NM} - \alpha_{FM} = \frac{g\,\mu_B}{4\pi M_S} g_{\uparrow\downarrow}^{T,eff} \frac{1}{t_{Co}} \qquad (2)$$

where μ_B is the Bohr magneton and the other quantities are defined in the main text. The calculated $g_{eff}^{\uparrow\downarrow}$ is then proportional to the generated 3D spin current density, namely:

$$J_S^{3D} = \frac{\hbar}{4\pi} g_{\uparrow\downarrow}^{T,eff} \left[\vec{M} \times \frac{d\vec{M}}{dt}\right] \qquad (3)$$

In a SP-FMR experiment the J_S^{3D} value depends on the measurement conditions, according to the following equation:

$$J_S^{3D} = \frac{Re\left(g_{eff}^{\uparrow\downarrow}\right)\gamma^2 h_{RF}^2 \hbar}{8\pi\alpha^2} \left(\frac{\mu_0 M_S - \sqrt{(\mu_0 M_S)^2 + 4\omega^2}}{(4\pi M_S\,\gamma)^2 + 4\omega^2}\right) \frac{2e}{\hbar} \qquad (4)$$

where h_{RF} is the strength of the oscillating magnetic field generated by the RF current and ω the RF frequency. For further details, see Ref.[6].

3. Alternative calculation to estimate the SCC efficiency according to the Inverse Spin Hall Effect (ISHE)

For the ISHE the spin current generated into the FM layer is pumped and converted in the bulk states of the spin-sink layer (in our case the Bi_2Te_3 layer). Here, the figure of merit of the SCC efficiency is the so-called

spin-Hall angle $\theta_{ISHE}^{\lambda_s}$= $\frac{J_{SP}}{\lambda_S tanh\,(\frac{t_{\mathrm{Bi2Te3}}}{2\lambda_S})J_S^{3D}}$, where λ_S is the spin diffusion length and t_{Bi2Te3} the thickness of the Bi_2Te_3 layer. J_S^{3D} is the spin current density generated in the SP experiment, which varies according to the frequency and the power of the RF current used to excite the system (see above in Supp. Info.) and which is, in our case, $3.28\ \cdot 10^5 Am^{-2}$. For a tentative calculation of the $\theta_{ISHE}^{\lambda_s}$, we consider $\lambda_S = 5\ nm$, a reasonable value among those found in literature for chalcogenide-based TIs.[7–10] As a result, we obtain $\theta_{ISHE}^{\lambda_s} = 0.088$, or alternatively a SCC efficiency around the 8.8 %.

4. XPS and RHEED measurements of the Si(111)/Sb_2Te_3/Bi_2Te_3 surface

In order to ensure the proper reconstruction of the Bi_2Te_3 surface upon the preparation of the sample to perform ARPES measurements, its chemical and structural evolution are followed by X-ray Photoemission Spectroscopy (XPS) and Reflection High-Energy Electron Diffraction (RHEED) characterization.

The preparation of the sample surface is performed in two steps: firstly, an Ar+ sputtering is carried out at 1.5 KeV and 10^5 mbar with a duration settled to 150 s, and subsequently an annealing process is performed for a complete reconstruction of the surface. In figure S2 the evolution of the XPS spectra for the unprocessed Si(111)/Sb_2Te_3/Bi_2Te_3 heterostructure (black line), after the sputtering treatment (red line) and upon the annealing (blue line), are shown. Moreover, to help the surface recover and to prevent the peeling of the Sb_2Te_3 and Bi_2Te_3 layers during the annealing, a small amount of Te flow (0.5 Å/s) is delivered to the chamber, being Te the most volatile element.

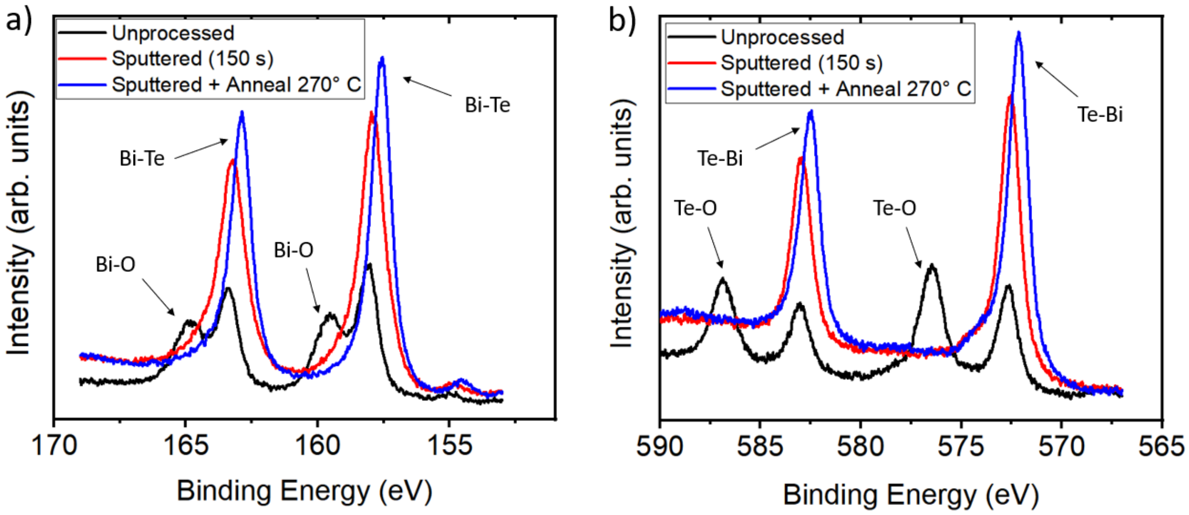

Figure S2. XPS spectra performed on the heterostructure Si/Bi2Te3/Sb2Te3, as it is (black line), after 150 s of Ar+ sputtering (red line) and after an annealing at 270 ° C (blue line). In panel (a) the energy of the 4f orbitals of Bi are shown, while panel (b) displays the 3d orbitals of Te.

In this case, the annealing temperature is decreased with respect to the Sb_2Te_3 and Bi_2Te_3 single layers studied in Ref.[11], which have been treated at 292 °C. By observing the XPS signals after the annealing (Fig. S2 blue

line), it can be seen that the weak shoulder belonging to the Bi-O (panel (a)) and Te-O (panel (b)) bonds disappear, thus confirming the full removal of the oxidized species.

In order to check the evolution of the crystallinity of the Si(111)/Sb_2Te_3/Bi_2Te_3 surface, RHEED measurements are performed after each treatment. As shown in Fig. S3 (a), RHEED does not reveal any electronics reflection pattern for the unprocessed sample, thus indicating that a continuous native oxide layer caps the whole surface. Differently, Figure S3 (b) shows a visible RHEED pattern by following the sputtering procedure, which

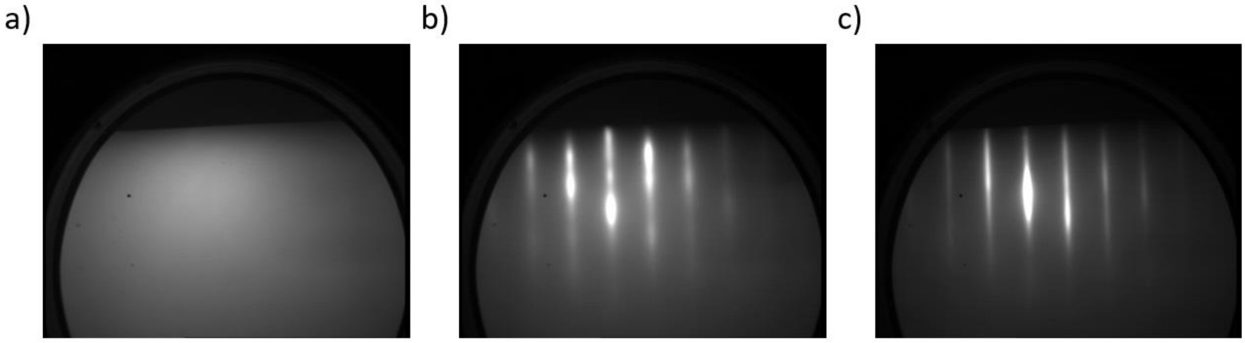

Figure S3. RHEED pattern for various stages of the surface processing: as deposited, in panel (a), after the 150 s Ar+ sputtering, in panel (b) and after sputtering and annealing, panel (c).

is however still not fully defined, indicating a partial damage of the surface crystalline structure. Finally, after the thermal annealing, the RHEED pattern becomes clearly defined, thus demonstrating an almost full reconstruction of the sample surface, as reported in in Fig. S3 (c).

Bibliography

1. Rimoldi, M. *et al.* Epitaxial and Large Area Sb2Te3 Thin Films on Silicon by MOCVD. *RSC Adv* **10**, 19936–19942 (2020).

2. Rimoldi, M. *et al.* Effect of Substrates and Thermal Treatments on Metalorganic Chemical Vapor Deposition-Grown Sb_2Te_3 Thin Films. *Cryst Growth Des* **21**, 5135–5144.

3. Kumar, A. *et al.* Large-Area MOVPE Growth of Topological Insulator Bi2Te3 Epitaxial Layers on i-Si(111) . *Cryst Growth Des* (2021) doi: 10.1021/acs.cgd.1c00328.

4. Tserkovnyak, Y., Brataas, A. & Bauer, G. E. W. Spin Pumping and Magnetization Dynamics in Metallic Multilayers. *Phys Rev B Condens Matter Mater Phys* **66**, 1–10 (2002).

5. Tserkovnyak, Y., Brataas, A. & Bauer, G. E. W. Enhanced Gilbert Damping in Thin Ferromagnetic Films. *Phys Rev Lett* **88**, 4 (2002).

6. Longo, E. *et al.* Large Spin-to-Charge Conversion at Room Temperature in Extended Epitaxial Sb2Te3 Topological Insulator Chemically Grown on Silicon. *Adv Funct Mater* **2109361**, (2021).

7. Jamali, M. *et al.* Giant Spin Pumping and Inverse Spin Hall Effect in the Presence of Surface and Bulk Spin-Orbit Coupling of Topological Insulator Bi2Se3. *Nano Lett* **15**, 7126–7132 (2015).

8. Dc, M. *et al.* Room-temperature High Spin–orbit Torque Due To Quantum Confinement in Sputtered BixSe(1–x) Films. *Nat Mater* **17**, 800–807 (2018).

9. Dc, M. *et al.* Observation of High Spin-to-Charge Conversion by Sputtered Bismuth Selenide Thin Films at Room Temperature. *Nano Lett* **19**, 4836–4844 (2019).

10. Dc, M. *et al.* Room-temperature Spin-to-charge Conversion in Sputtered Bismuth Selenide Thin Films Via Spin Pumping From Yttrium Iron Garnet. *Appl Phys Lett* **114**, (2019).

11. Locatelli, L. *et al.* Magnetotransport and ARPES Studies of the Topological Insulators Sb2Te3 and Bi2Te3 Grown by MOCVD on Large-area Si Substrates. *Sci Rep* **12**, (2022). doi:10.1038/s41598-022-07496-7
